# Assessment of image quality and detectability in computed tomography: Inter‐laboratory comparison of Fourier‐based metrics

**DOI:** 10.1002/acm2.70838

**Published:** 2026-10-06

**Authors:** Pascal Monnin, Damien Racine, Luis Ammour, Yves Barbotteau, Davide Cester, Dimitar Petrov, Kwinten Torfs, Raffaele Villa, Nicoletta Paruccini, Joël Greffier, Anaïs Viry

**Affiliations:** ^1^ Institute of radiation physics (IRA) Lausanne University Hospital (CHUV) and University of Lausanne Lausanne Switzerland; ^2^ CHU Nantes Nantes Université Nantes France; ^3^ Service d'imagerie‐Hôpital Clairval Ramsay Santé Marseille France; ^4^ Diagnostic and Interventional Radiology University Hospital Zurich University of Zurich Zürich Switzerland; ^5^ Department of Imaging and Pathology KU Leuven Leuven Belgium; ^6^ Medical Physics Department Fondazione IRCCS San Gerardo dei Tintori Monza Italy; ^7^ IMAGINE UR UM 103 Department of Medical Imaging Nîmes University Hospital Montpellier University Nîmes France

**Keywords:** computed tomography, detectability, Fourier‐based metrics, image quality, inter‐laboratory comparison

## Abstract

**Background:**

The task‐based assessment of image quality in computed tomography (CT) is increasingly used for benchmarking, optimizing acquisition and reconstruction parameters, and predicting observers’ performance. To achieve consistent and reproducible results rely on standardized methodologies and guidelines.

**Purpose:**

This study performed an inter‐laboratory comparison to evaluate the consistency of CT image quality metrics and assess inter‐laboratory variability.

**Methods:**

Six series of images of a Catphan 500 phantom were acquired at three dose levels (1.5/5.0/15.0 mGy) with two (standard and high‐resolution) kernels and sent to seven independent laboratories. Participants calculated the task transfer function (TTF) for spatial resolution and noise power spectrum (NPS) for noise magnitude and texture using standardized Fourier‐based analysis on the shared images. The TTFs and NPS were used in the non‐prewhitening observer model without (NPW) and with eye filter (NPWE) to calculate a detectability index for small (2 mm) and large targets (10 mm).

**Results:**

The difference observed in TTFs resulted mainly from the different noise reduction techniques used during the calculation. The frequency corresponding to the TTF value 50% varied from 0.27 to 0.43 mm^−1^ for the standard images and from 0.73 to 0.92 mm^−1^ for the high‐resolution images. Variability in NPS primarily stemmed from the choice of regions of interest location in non‐stationary noise images that lead to magnitude differences up to 40% for the standard kernel and 70% for the high‐resolution kernel. The combined differences in TTF and NPS resulted in differences up to 39% in the NPW detectability index. The large variability in the human visual transfer function and visualization parameters increased the differences by a factor of 6.25 in the NPWE model and highlighted the lack of standardization.

**Conclusion:**

Adapting TTF and NPS to a more complex and localized analysis of CT images with non‐uniform CNRs, rather than a simple global evaluation, increases computational variability and requires a move towards more standardization of metrics. In the absence of complete standardization of image quality measurement, documentation of methods and all calculation parameters is essential for more robust evaluation of CT image quality.

## 1 INTRODUCTION

Task transfer function (TTF) and noise power spectrum (NPS) are advanced CT metrics used to evaluate spatial resolution and noise under realistic imaging conditions. TTF assesses system resolution at specific contrast levels, considering contrast‐to‐noise ratio (CNR) of clinical images, while NPS evaluates noise magnitude and texture. Together, they allow the computation of a detectability index (*d′*) for a specific task object using mathematical model observers.^1^, ^2^, ^3^ These Fourier‐based metrics are of great importance to benchmark image quality across CT systems or optimize acquisition and reconstruction parameters to improve dose efficiency.^4^, ^5^, ^6^, ^7^ These measurements are critical for comprehensive CT image quality assessment, especially for evaluating the performance of modern reconstruction algorithms like iterative reconstruction (IR) or deep learning image reconstruction (DLIR), and detectability.^8^, ^9^, ^10^, ^11^, ^12^

Significant standardization efforts have been made for TTF and NPS measurements in CT, primarily driven by the American Association of Physicists in Medicine (AAPM).^13^ These efforts aim to provide consistent, objective, and standardized quality assurance procedures.^14^, ^15^, ^16^, ^17^, ^18^ The AAPM has commissioned task groups, such as TG169 and TG233, to formalize calculation guidelines for TTF and NPS to establish a common methodology for measuring spatial resolution and noise. Standardized, open‐source, or vendor‐neutral software have been developed to automate the measurement of TTF and NPS across various phantoms (e.g. AAPM CT Performance phantom, Catphan, ACR 464, Mercury v4.0, …) with the aim of harmonization and reducing human variability and analysis time. Researchers have therefore standardized circular‐edge techniques for in‐plane TTF and computation methods for NPS, including their adaptation to assess task‐based detectability.^19^, ^20^, ^21^, ^22^, ^23^ These methods are evolving to address non‐linear, non‐stationary characteristics of modern IRs and DLIRs, adapting TTF and NPS to a more complex and localized analysis in images with non‐uniform CNRs. These evolutions allow for more robust evaluation of CT image quality, moving from global assessments to task‐based metrics.^24^, ^25^, ^26^

Nevertheless, there is still a recognized lack of universal standardization in TTF and NPS measurements in CT.^27^, ^28^, ^29^ Despite the existence of guidelines such as those of AAPM Task Group 233, significant variability persists in how these metrics are applied across different clinical tasks in different laboratories. The key aspects of this difficulty include nonlinear reconstruction effects of IR and DLIR algorithms, with TTF and NPS potentially varying depending on object contrast and dose, making measurement more complex. TTF measurements also depend heavily on the edge and calculation methods used, and NPS calculation requires large areas, which can be inconsistent with local noise assessment.^30^, ^31^, ^32^, ^33^ To address this variability, there is a shift toward automated computation software and, in some cases, extending these measurements from phantoms directly to clinical images to provide a more task‐based, in vivo assessment.^34^, ^35^

Despite these efforts, no study has yet systematically quantified the inter‐laboratory variability arising from the diversity of computational implementations when applied to a common CT image dataset. Pioneering intercomparison of MTF and NPS measurement methods in digital radiography across multiple implementations have revealed significant method‐dependent differences.^36^, ^37^ However, these studies predated the widespread adoption of IR and DLIR algorithms and the formalization of TTF and NPS within a task‐based detectability framework. Intercomparison of physical metrics (TTF, NPS, *d′*) is useful to develop reliable objective task‐based and observer‐based methods to ensure results consistency and comparability between different studies. It should also ensure that image quality assessment tools and automated quality control procedures are reliable across different imaging environments.^38^, ^39^ In this work, we report on an inter‐laboratory comparison (ILC) among seven different laboratories from four different countries that calculated TTF and NPS for six series of images with different resolution/noise tradeoffs. The objective of this work was to quantify inter‐laboratory variability in TTF, NPS, and *d'* calculations for the NPW and NPWE model observers.

## 2 METHODS

### 2.1 Data acquisition and reconstruction

A Catphan 500 phantom (The Phantom Laboratory, Salem, NY, USA) was imaged on a GE Revolution Apex CT system (GE Healthcare, Milwaukee, MI, USA) in helical mode with a tube voltage of 120 kVp, a pitch factor of 0.984, a beam collimation of 64 × 0.625 mm, a scan field‐of‐view (FOV) of 300 mm (Small Body SFOV setting) and a rotation time of 0.5 s. Three tube currents were selected (45/145/450 mA) to target three volume CT dose index (CTDI_vol_) values: 1.5, 5.0 and 15.0 mGy (32 cm CTDI phantom). Axial slices were reconstructed with a pixel size of 0.488 mm (reconstructed FOV 250 mm—matrix 512 × 512) and a slice thickness / interval of 1.25 / 0.625 mm using the partial model‐based iterative reconstruction algorithm ASIR‐V at 50%. The phantom acquired at the three dose levels was reconstructed using a standard (ST) kernel (STD) and a high‐resolution (HR) kernel (Bone+), giving six series of images for analysis denoted ST1, ST5, ST15, HR1, HR5 and HR15 (Table 1). The acquisition of the phantom was repeated five times by series and provided 160 images by series to calculate TTF and 380 images by series to calculate NPS.

**TABLE 1:** Series of images.

Series of images	ST1	ST5	ST15	HR1	HR5	HR15
**Acquisition**						
CTDI_vol_ [mGy]	1.5	5.0	15.0	1.5	5.0	15.0
Kernel	STD	STD	STD	Bone+	Bone+	Bone+
**TTF**						
Rod material	LDPE	LDPE	LDPE	PTFE	PTFE	PTFE
Contrast [HU]	200	200	200	800	800	800
CNR_T_	89	147	200	75	133	225
**Model observer**						
Object diameter [mm]	10.0	10.0	10.0	2.0	2.0	2.0

Abbreviations: HU indicates Hounsfield units; TTF indicates task‐based transfer function; CNR_T_ indicates the total effective contrast‐to‐noise ratio; CTDI_vol_ indicates volume computed tomography dose index.

### 2.2 Task transfer function (TTF)

The laboratories had to measure the TTFs on two 13.7 mm diameter rods located on the CTP401 Module (slice width, sensitometry and pixel size) of the phantom. The low‐contrasted LDPE rod (Low Density Polyethylene ≈ ‐100 HU at 120 kVp, contrast with the phantom background 200 HU) was used for the three ST image series. The high‐contrasted PTFE rod (Polytetrafluoroethylene, Teflon® ≈ 900 HU at 120 kVp, contrast with the phantom background 800 HU) was used for the three HR image series (Table 1).

To secure a reliable estimate of the TTF, physics guidelines such as those from the AAPM^21^, ^30^ recommend a specific minimum total effective CNR (CNR_T_). The CNR_T_ of TTF computation was measured from the sum of all the available images sent to the laboratories. CNR_T_ is the difference in mean HU value between the rod (${\bar{HU}}_{s,rod}$) and its background (${\bar{HU}}_{s,bgd})$, divided by the standard deviation of the background noise ($\sigma_{s,bgd}$) in the summed image, as indicated in Equation (1).

(1) $$CNR_{T}=\frac{\left|{\bar{HU}}_{s,rod}-{\bar{HU}}_{s,bgd}\right|}{\sigma_{s,bgd}}$$

The CNR_T_ value must be considered as the maximal CNR available for TTF computation, which may be higher than the value used by laboratories if not all images have been used for calculation.

Each laboratory had the free choice of its specific implementation of the method used for TTF calculation, and had to provide information on the options chosen, in particular edge spread function (ESF) or line spread function (LSF) conditioning, symmetrization or fitting (Table 2).

**TABLE 2:** Calculation parameters used by the laboratories.

Laboratory	L1	L2	L3	L4	L5	L6	L7
**Calculation software**	Home‐made	Home‐made	iQMetrix‐CT v1.2	iQMetrix‐CT v1.2	Home‐made	Imquest‐based	Home‐made
**ESF / LSF**							
Monotonic regression	No	1/pixel	No	No	10/pixel	No	10/pixel
Windowing	No	Hann	No	No	Poisson	Hann	No
Symmetrization	No	No	Yes	No	No	No	No
Analytical fit	No	LSF tails	ESF	ESF	No	No	ESF
**NPS**							
Windowing	No	No	No	No	No	No	No
Background subtraction	Poly 2D	Poly 2D	Poly 2D	Poly 2D	No	Im. subtr.	Poly 2D
Number of ROIs	1	^(^ ^*^ ^)^	8	4	1	8	8
ROIs size [pixels]	64 × 64	39 × 39	48 × 48	86 × 86	200 × 200	64 × 64	64 × 64
**VTF**							
Reference	Solomon (2016)	Burgess (1994)	Eckstein (2003)	Eckstein (2003)	Gang (2011)	Saunders (2006)	Eckstein (2003)
Parameter n	3.0	1.3	3.0	3.0	1.0	3.0	3.0
Parameter c	6.44 deg^−1^	4.06 mm^−1^	1.96 deg^−1^	1.96 deg^−1^	2.20 mm^−1^	6.44 mm^−1^	1.96 deg^−1^
Parameter a	0.68	2.00	0.68	0.68	1.00	0.68	0.68
Visualization distance [mm]	400	600	500	500	500	500	400
Display magnification	1.00	3.00	3.00	3.00	1.00	1.37	1.20

*Note*: (*) dependent on the image.

### 2.3 Noise power spectrum (NPS)

The laboratories had to measure the NPS on the images of the CTP486 Module (uniformity) of the phantom. This module is cast from a uniform material of CT number designed to be within 2% of water's density. The NPS were measured for the six series of images on regions of interest (ROIs) freely chosen by the laboratories (number, position and size). Each laboratory had the free choice of its specific implementation of the method used for NPS calculation and had to provide information on the chosen options for the position, number and size of the ROIs, and for an eventual detrending methods (background subtraction techniques such as 2D polynomial or mean image subtraction) used to subtract out low‐frequency trends in the spatial domain (Table 2).

### 2.4 Detectability index

The laboratories had to calculate a detectability index (*d′*) for a large disc (diameter 10.0 mm) for the three series of ST images and a small disc (diameter 2.0 mm) for the three series of HR images (Table 1). The disc of radius *R* could be calculated from simulation of the object or by using the analytical Fourier transform of a 2D uniform function $S_{obj}\left(R,f_{x},f_{y}\right)$ with the Bessel function of the first kind of the first order J_1_ (Equation (2)).

(2) $$S_{obj}\left(R,f_{x},f_{y}\right)=\frac{R}{\sqrt{f_{x}^{2}+f_{y}^{2}}}\cdot J_{1}\left(2\pi R\sqrt{f_{x}^{2}+f_{y}^{2}}\right)$$

In the first step, *d′* by object contrast unit ($d^{\prime }/C$ expressed in HU^−1^) were calculated using the NPW (non prewhitening) model observer with the measured TTFs and NPS:

(3) $$\begin{array}{cc} & \frac{d_{NPW}^{\prime }}{C}=\\ & \frac{\int \;\;\;\int TTF_{xy}^{2}\left(f_{x},f_{y}\right)\cdot S_{obj}^{2}\left(R,f_{x},f_{y}\right)\;df_{x}df_{y}}{\sqrt{\int \;\;\;\int TTF_{xy}^{2}\left(f_{x},f_{y}\right)\cdot NPS_{xy}\left(f_{x},f_{y}\right)\cdot S_{obj}^{2}\left(R,f_{x},f_{y}\right)\;df_{x}df_{y}}} \end{array}$$

To mimic how a human observer detects signals (like tumours or lesions) in noisy images, the NPW model was enhanced with an “eye filter” to better reflect human vision. The laboratories had to compute the $d^{\prime }/C$ values using the NPWE (non prewhitening with eye filter) model described by Equation (4).

(4) $$\frac{d_{NPWE}^{\prime }}{C}=\frac{\int \;\;\;\int VTF^{2}\left(f_{x},f_{y}\right)\cdot TTF_{xy}^{2}\left(f_{x},f_{y}\right)\cdot S_{obj}^{2}\left(R,f_{x},f_{y}\right)df_{x}df_{y}}{\sqrt{\int \;\;\;\int VTF^{4}\left(f_{x},f_{y}\right)\cdot TTF_{xy}^{2}\left(f_{x},f_{y}\right)\cdot NPS_{xy}\left(f_{x},f_{y}\right)\cdot S_{obj}^{2}\left(R,f_{x},f_{y}\right)df_{x}df_{y}}}$$

The laboratories had to use a visual transfer function (VTF) of the human eye expressed by the general form given in Equation (5).^14^, ^40^, ^41^

(5) $$VTF\left(u\right)=u^{n}\cdot exp\left(-cu^{a}\right)$$

where *u* is the spatial frequency in degree^−1^ seen by the observer:

(6) $$u=\frac{\pi }{180}\cdot \frac{D}{M}\cdot \sqrt{f_{x}^{2}+f_{y}^{2}}$$

where the display magnification *M* is the ratio between the image size on the display screen and the image FOV and *D* is the observer's distance from the display screen. Each laboratory had the free choice to choose the parameters *a*, *c* and *n* of the VTF, the display magnification *M* and the observer‐to‐screen distance *D*.

### 2.5 Statistical analysis

The root mean square deviation (RMSD) was used to quantitatively measure the average distance between the TTFs and NPS computed by the laboratories and the median TTF and NPS for each of the six series, to assess similarity. It was calculated as the square root of the average of squared differences, according to Equations (7) and (8).

(7) $$RMSD_{TTF}=\sqrt{\frac{1}{f_{Nyq}}\cdot \int_{0}^{f_{Nyq}}{\left(TTF\left(f\right)-\tilde{TTF}\left(f\right)\right)}^{2}df}$$

(8) $$RMSD_{NPS}=\sqrt{\frac{1}{f_{Nyq}}\cdot \int_{0}^{f_{Nyq}}{\left(NPS\left(f\right)-\tilde{NPS}\left(f\right)\right)}^{2}df}$$

where the symbol ∼ denotes the median value, $f=\sqrt{f_{x}^{2}+f_{y}^{2}}$ is the radial frequency and $f_{Nyq}$ is the radial Nyquist frequency determined from the pixel spacing Δx, according to Equation (9).

(9) $$f_{Nyq}=\frac{\sqrt{2}}{2\cdot \Delta x}$$

Equations (7) and (8) determine the deviation between the TTF or NPS curves calculated by the laboratories and their corresponding median spectrum. Using 1D integrals over the radial frequency *f* (without a Jacobian) amounts to analyzing the TTF or NPS as one‐dimensional mathematical functions in which each frequency carries equal weight, regardless of the geometric contribution of each frequency band to the final image. A lower RMSD value indicates higher similarity, while a higher value indicates greater deviation.

The average NPS frequency, denoted *f_NPS_*, was used to quantify the fineness of the measured noise:

(10) $$f_{NPS}=\frac{\int NPS\left(f\right)\cdot f\;df}{\int NPS\left(f\right)\;df}$$

Relative variations of $d^{\prime }/C$ values obtained at 1.5 and 5.0 mGy compared to those at 15.0 mGy (ST1/ST15, ST5/ST15, HR1/HR15, HR5/HR15) were calculated to compare relative $d^{\prime }/C$ differences between laboratories due to dose variations without significant change in the frequency content (resolution) of the images.

## 3 RESULTS

Seven independent laboratories (L1 to L7) took part in the ILC and calculated the TTF, NPS and *d′* for the six series of images. L1, L2, L5 and L7 used home‐made software. L3 and L4 used the iQMetrix‐CT v1.2 software developed by a working group from the French Society of Medical Physicists (SFPM),^22^ and L6 performed a semi‐automated calculation with the open‐source imQuest software (Duke University, Durham, NC, USA).^21^

### 3.1 Task‐based transfer function (TTF)

The CNR_T_ available for TTF computation varied between 75 and 225 for the six series of images, largely above the minimum value of 15 required to achieve an error less than 10% in the TTF^21^, ^30^ (Table 1). The seven laboratories chose different calculation options with different data processing techniques (Table 2). Four out of seven laboratories used ESF / LSF conditioning methods: ESF monotonic regression which decreases the impact of noise on the TTF and make it monotonic, and/or LSF windowing which multiplies the LSF by a smooth window function (such as a Hann window) before performing the Fourier transform to force the outer tails of the LSF curve to smoothly descend to zero, reduce truncation artifacts and minimize spectral leakage. L2 and L6 applied a smooth Hann window to the LSF and L5 a Poisson window. Labs L5 and L7 finely resampled the ESF with a regular 0.1‐pixel pitch and L7 additionally applied a median filtering over a moving 0.1‐pixel window on the ESF. In contrast, the TTF from L2 was computed from an ESF that was interpolated with a sampling spacing equal to 100% the pixel pitch. In iQMetrix and ImQuest software, TTF conditioning can be either de‐activated, manually activated or automatically activated when CNR_T_ is lower than 15. Therefore, laboratories L3 and L4 did not use conditioning options. Laboratories L3, L4 and L7 adjusted an analytical fit to the ESF, two Gaussian functions added to a sigmoid for L3 and L4, and a Gaussian window added to an error function (erf) for L7. L2 fitted the LSF tails with a linear function which was subtracted from the LSF to remove noise. Only L3 corrected the ESF asymmetry caused by different scattered radiation amounts between the rod and the phantom background.

The TTFs of the three series ST showed a low‐pass shape (Figure 1 ST1‐ST5‐ST15). The frequency at the TTF value 50% (*f_50_*) varied from 0.27 to 0.43 mm^−1^. The RMSD with the median of the seven TTFs (RMSD_TTF_, Table 3) showed that differences in TTFs were particularly notable at low dose (ST1 series) for L2 and L7 that yielded TTFs shifted towards lower frequencies.

**FIGURE 1:**
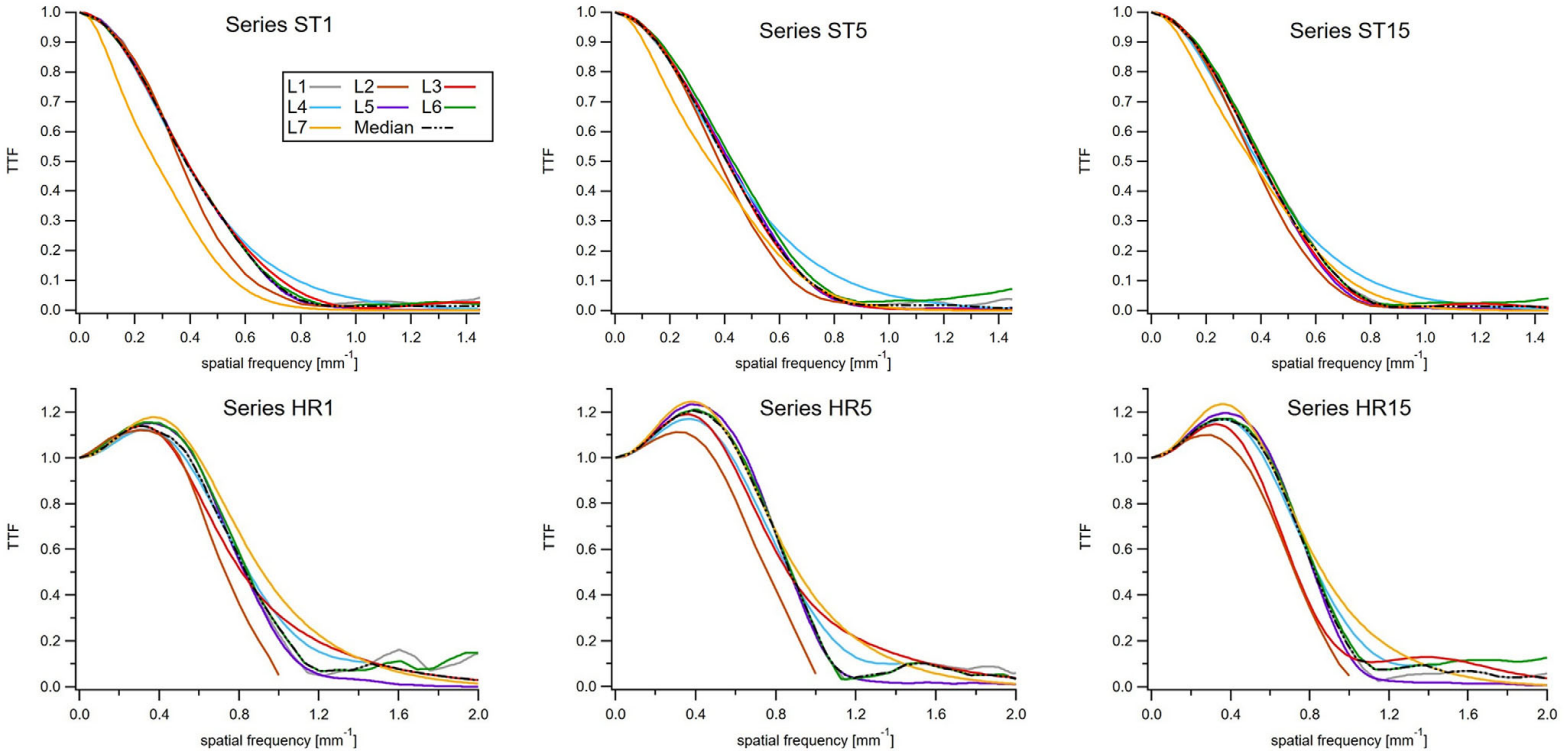
TTFs calculated by the seven laboratories for the six series of images.

**TABLE 3:** Root mean square deviation with the median TTF (RMSD_TTF_), TTF frequency at 50% (*f_50_*) and maximum TTF value (TTF_max_).

Lab	L1	L2	L3	L4	L5	L6	L7
**RMSD**_**TTF**_							
Series ST1	0.44	4.22	1.28	1.14	0.39	0.49	12.8
ST5	0.39	3.60	0.33	0.35	0.81	2.30	6.31
ST15	1.51	2.89	0.24	1.29	0.54	1.40	4.35
HR1	3.61	67.7	5.60	4.38	3.61	3.79	8.50
HR5	1.38	76.5	7.86	5.66	3.66	0.89	7.58
HR15	1.05	42.7	9.12	4.27	3.25	3.68	6.94
**f**_**50**_ **[mm** ^**−1**^ **]**							
Series ST1	0.38	0.37	0.39	0.38	0.38	0.39	0.27
ST5	0.41	0.38	0.41	0.41	0.42	0.43	0.35
ST15	0.41	0.37	0.40	0.39	0.40	0.41	0.37
HR1	0.83	0.73	0.81	0.85	0.83	0.84	0.92
HR5	0.88	0.76	0.86	0.87	0.87	0.88	0.91
HR15	0.83	0.73	0.73	0.84	0.82	0.84	0.87
**TTF**_**max**_							
Series HR1	1.13	1.12	1.14	1.12	1.15	1.15	1.18
HR5	1.21	1.11	1.19	1.17	1.23	1.21	1.25
HR15	1.17	1.10	1.15	1.17	1.20	1.17	1.23

The TTFs of the series HR showed edge‐enhancement (overshoot at low spatial frequency, Figure 1 HR1‐HR5‐HR15) and only residual noisy tails below 10% above the Nyquist frequency (1.45 mm^−1^) considered for TTF and RMSD calculation (Equation (9)). The RMSD with the median of the seven TTFs (RMSD_TTF_, Table 3) showed that differences in TTFs were globally higher for the HR than for the ST series, with a notable shift towards lower frequencies for L2 and L3 at 15 mGy (*f_50_* between 0.73 and 0.76 mm^−1^). The other laboratories gave higher *f_50_* values, between 0.81 and 0.92 mm^−1^. The maximum value (overshoot) of the TTF varied between 1.10 and 1.25 at frequencies between 0.30 and 0.40 mm^−1^. It is of note that L3 and L4 used the same computational software (iQMetrix‐CT v1.2) but the (optional) ESF symmetrization resulted in different TTFs.

### 3.2 Noise power spectra (NPS)

The seven laboratories chose different NPS calculation options with different ROI numbers and positions in the images (Table 2 and Figure 2). No laboratory used windowing methods which are applied to ROIs before the Fourier transform to mitigate spectral leakage caused by non‐periodic signal boundaries. All laboratories, apart from L5, used background noise suppression methods. Laboratories L1, L2, L3, L4 and L7 subtracted a first‐ or second‐order 2D polynomial fit to the image data. By subtracting two consecutively acquired, identical images (or an average background), L6 eliminated low‐frequency noise from the images before NPS calculation, leaving only random noise. These two methods provided similar results while NPS from L5 consequently strongly increased at very low‐frequency, below 0.05 mm^−1^ (Figure 3).

**FIGURE 2:**
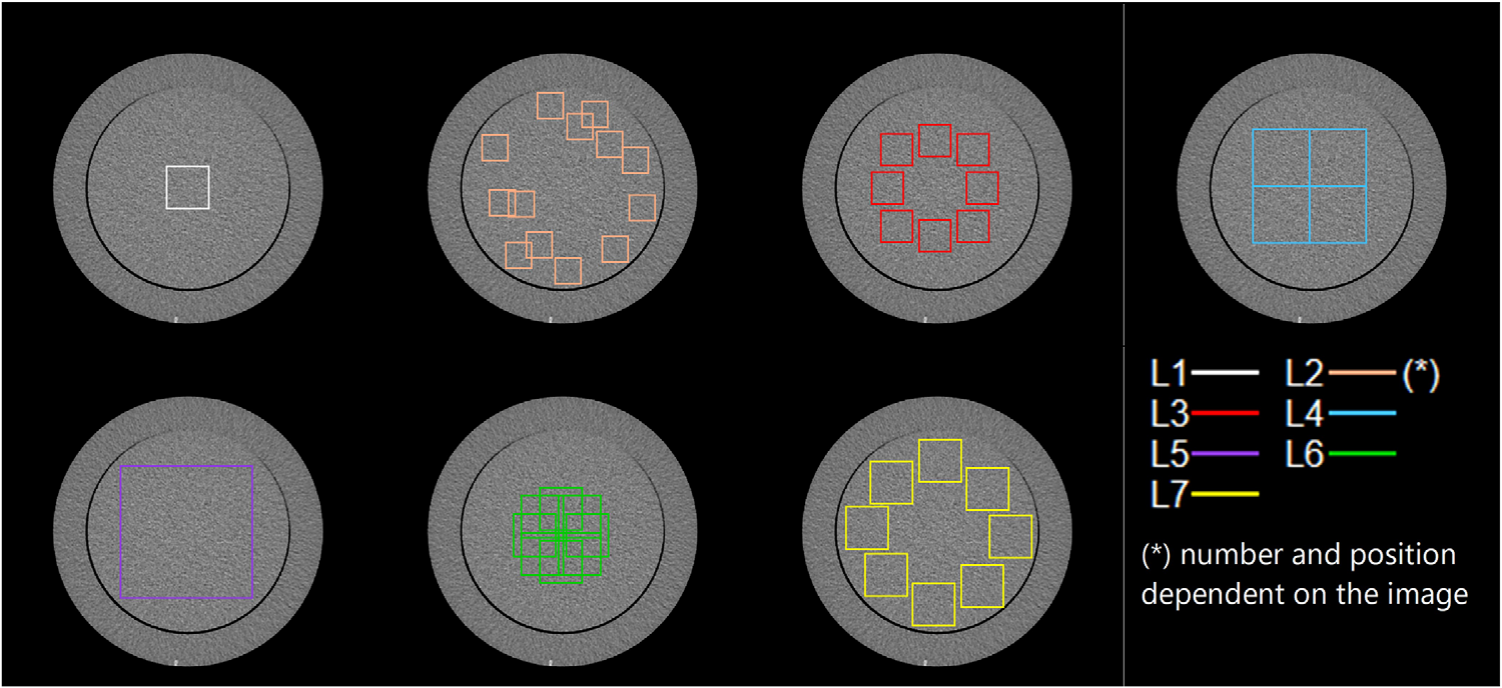
Region of interests (ROIs) used for NPS measurement by the seven laboratories for the six series of images.

**FIGURE 3:**
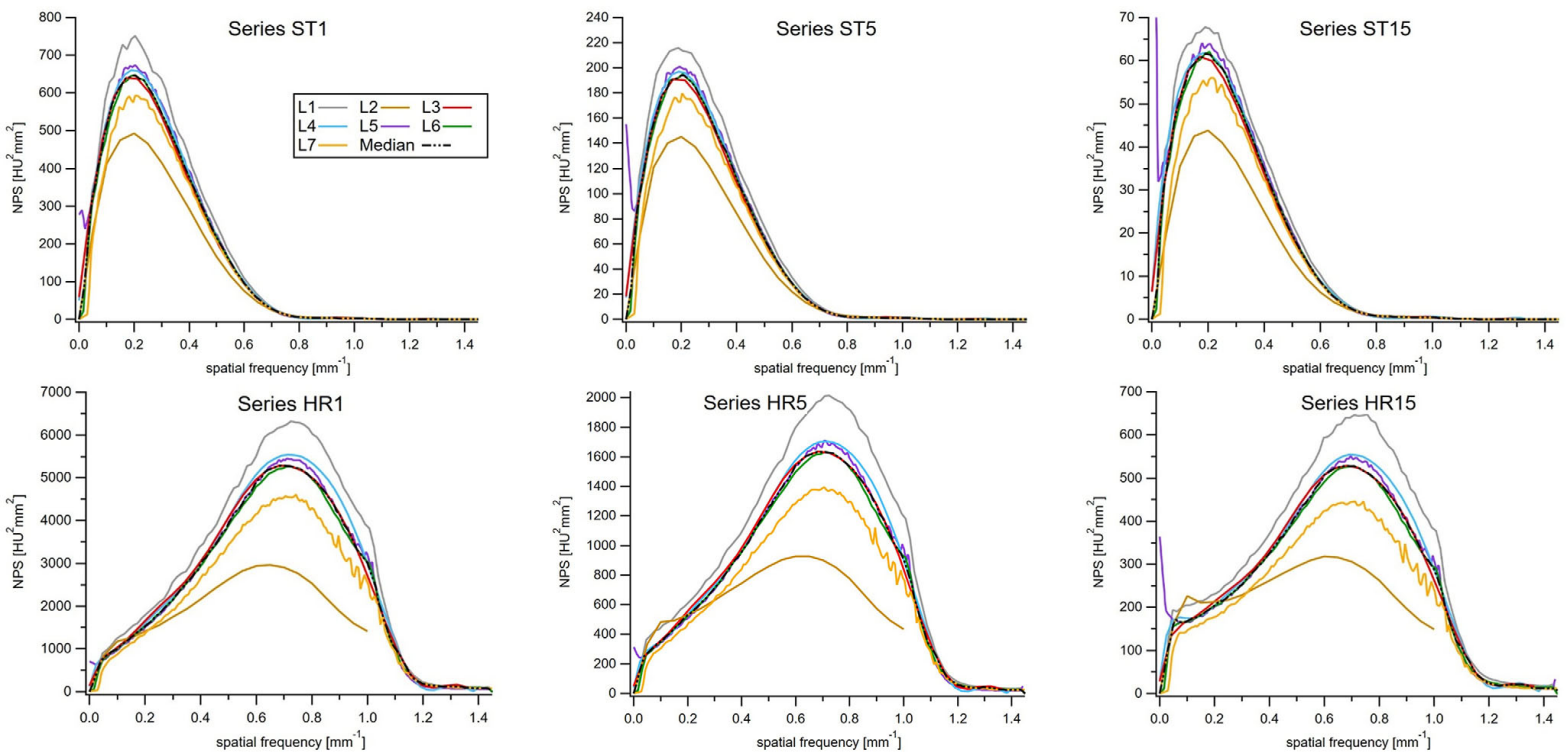
NPS calculated by the seven laboratories for the six series of images.

The RMSD with the median of the seven NPS (RMSD_NPS_) show that differences in NPS were the highest for L2 and L7 for the HR kernel (Table 4). NPS for L3, L4, L5 and L6 were similar and close to the median NPS for both the ST and HR kernels while NPS from L1 were systematically higher and NPS from L2 and L7 systematically lower. NPS from L1 and L7 showed differences in magnitude only (*NPS_max_*), while NPS from L2 showed differences in magnitude and shape (*f_NPS_*). The maximum NPS value showed maximum differences of 40% for the ST series and 70% for the HR series. The index *f_NPS_* was close for all the laboratories for the ST kernel, between 0.27 and 0.29 mm^−1^, more variable for the HR kernel, between 0.53 and 0.66 mm^−1^ (Table 4).

**TABLE 4:** Root mean square deviation with the median NPS (RMSD_NPS_), average NPS frequency *f_NPS_* and maximum NPS value (NPS_max_).

Lab	L1	L2	L3	L4	L5	L6	L7
**RMSD**_**NPS**_ **(x 10** ^**−2**^ **) [HU** ^**2**^ **mm** ^**2**^ **]**							
Series ST1	4.61	8.11	1.43	1.57	4.18	0.72	4.25
ST5	1.34	2.61	0.44	0.53	2.04	0.21	1.35
ST15	0.37	0.97	0.16	0.23	2.18	0.07	0.47
HR1	58.0	134.8	8.71	18.3	11.1	6.22	45.8
HR5	20.6	41.2	2.31	4.77	4.30	2.17	15.3
HR15	6.67	12.4	0.89	1.85	4.12	0.71	5.32
**f**_**NPS**_ **[mm** ^**−1**^ **]**							
Series ST1	0.28	0.28	0.27	0.27	0.27	0.28	0.29
ST5	0.28	0.28	0.27	0.27	0.27	0.28	0.29
ST15	0.28	0.28	0.27	0.27	0.27	0.28	0.29
HR1	0.66	0.57	0.64	0.65	0.65	0.65	0.65
HR5	0.65	0.55	0.64	0.64	0.64	0.64	0.64
HR15	0.64	0.53	0.62	0.63	0.61	0.63	0.62
**NPS**_**max**_ **[HU** ^**2**^ **mm** ^**2**^ **]**							
Series ST1	752	493	640	660	673	647	593
ST5	216	145	191	197	201	194	180
ST15	68	44	61	62	64	62	56
HR1	6328	2960	5283	5544	5450	5266	4611
HR5	2015	928	1635	1704	1711	1632	1394
HR15	647	318	529	554	551	528	446

### 3.3 Detectability index

A detectability index *d′* was calculated using the NPW observer model with the TTFs and NPS provided by the seven laboratories. The differences in TTF and NPS added up in the NPW model. The *d′* values were systematically higher for the series ST compared to the series HR (Table 5 and Figure 4). The *d′/C* values varied by 39% (1.24 – 1.85 HU^−1^) between the laboratories for the series ST15 and by 17% (0.109 – 0.130 HU^−1^) for the series HR15. The relative variations of *d′/C* values obtained at 1.5 and 5.0 mGy compared to those at 15.0 mGy (ST1/ST15, ST5/ST15, HR1/HR15, HR5/HR15) showed differences up to 8.2% between laboratories (Figure 5).

**TABLE 5:** *d′* values for the NPW and NPWE observer models.

Lab	L1	L2	L3	L4	L5	L6	L7
**NPW d′/C [HU** ^**−1**^ **] (x 10** ^**−2**^ **)**							
Series ST1	45.7	54.1	45.6	45.0	44.0	48.0	57.6
ST5	79.7	100.0	82.6	80.1	77.3	86.7	103.3
ST15	138.5	184.7	143.2	135.2	124.1	150.2	183.5
HR1	3.74	4.68	3.96	3.95	4.08	4.11	4.35
HR5	6.62	7.70	7.04	7.01	7.28	7.20	7.83
HR15	10.9	11.8	11.4	11.5	11.8	11.8	13.0
**NPWE d′/C [HU** ^**−1**^ **] (x 10** ^**−2**^ **)**							
Series ST1	33.3	15.4	4.79	5.00	21.3	7.35	8.60
ST5	57.8	28.4	8.70	10.2	38.7	14.5	16.5
ST15	100.3	51.8	15.5	16.8	68.7	25.2	30.2
HR1	2.75	3.55	0.63	0.61	3.43	2.09	2.23
HR5	4.62	5.85	1.24	1.14	6.20	3.77	4.08
HR15	6.83	9.65	2.26	1.98	10.3	6.49	6.99

**FIGURE 4:**
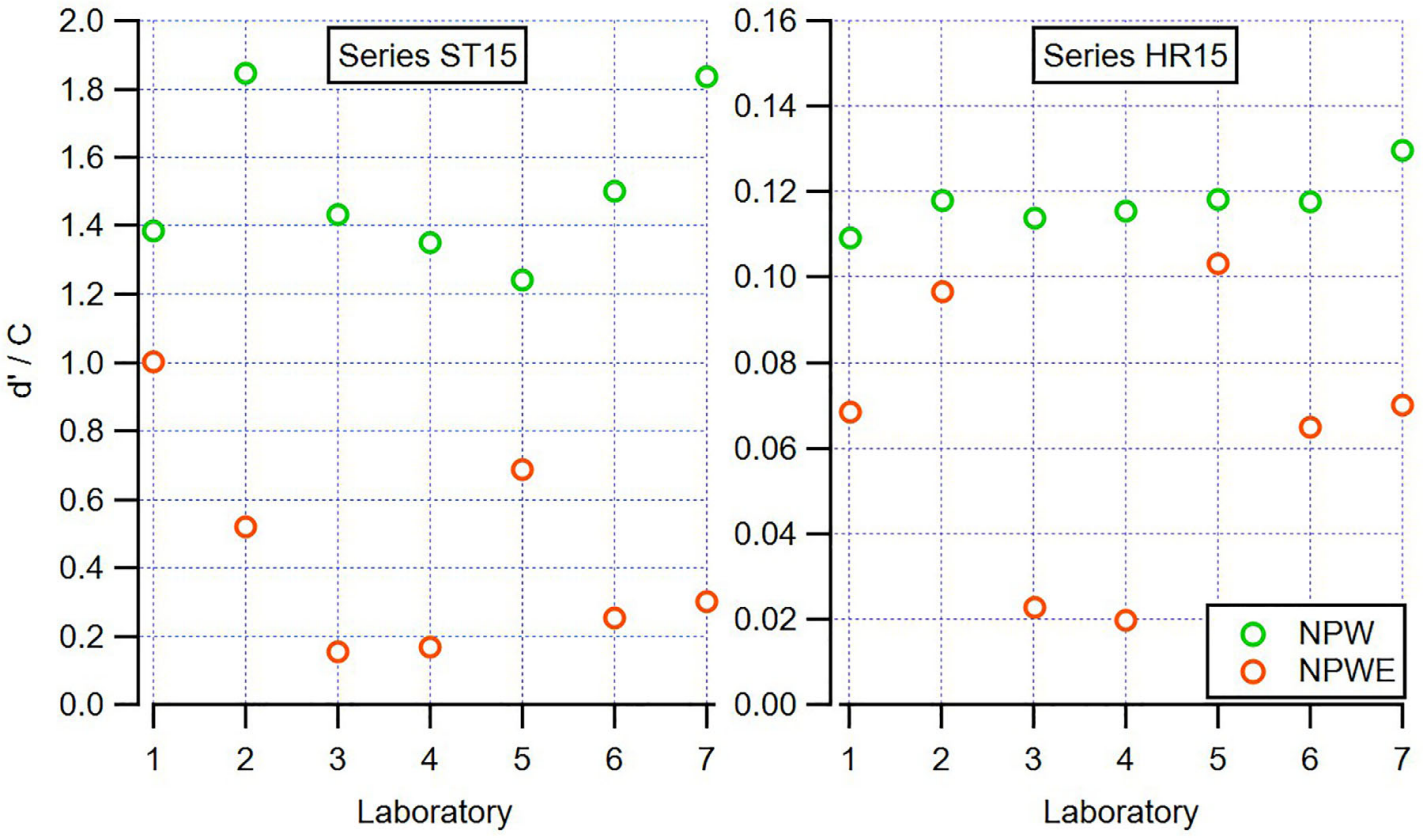
Detectability index by contrast unit (d′/C) of the NPW/NPWE models for the series ST15 and HR15.

**FIGURE 5:**
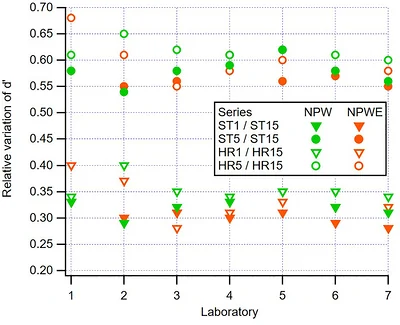
Relative variation of detectability indices *d′* obtained at 1.5 and 5.0 mGy compared to 15.0 mGy for the NPW/NPWE models.

The laboratories chose five different VTFs with visualization distances ranging from 400 to 600 mm and display magnifications between 1.0 and 3.0 in the NPWE observer model (Table 2). Apart from L1, all the laboratories chose VTFs whose peak between 3.5 and 4.0 deg^−1^ that match human vision (Figure 6).^40^ Given these differences, the VTF expressed in linear frequency (mm^−1^) showed considerable variations in shape, mode (peak between 0.08 and 0.80 mm^−1^) and frequency bandwidth. These differences dramatically added up to a variability in *d′/C* values that reached at 15 mGy a factor 6.45 for the series ST (0.155 – 1.00 HU^−1^) and a factor 5.15 for the series HR (0.020 – 0.103 HU^−1^) (Table 5). The relative variations of *d′/C* values obtained at 1.5 and 5.0 mGy compared to those at 15.0 mGy (ST1/ST15, ST5/ST15, HR1/HR15, HR5/HR15) showed differences up to 9.5% between laboratories (Figure 6).

**FIGURE 6:**
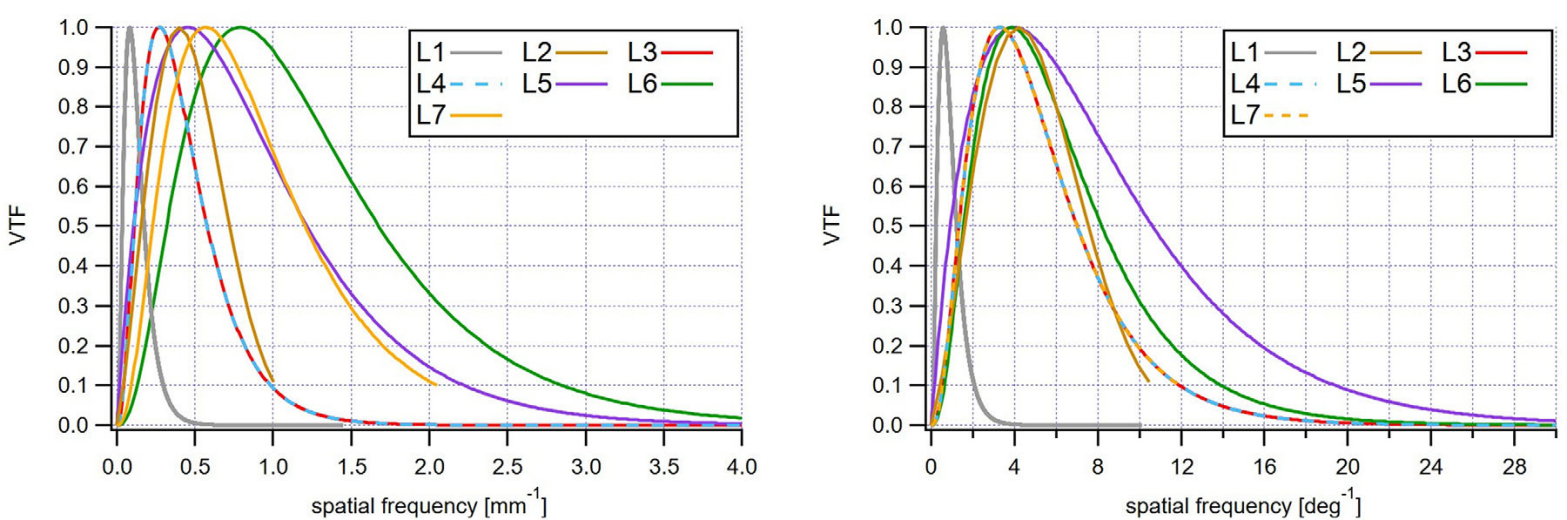
VTFs as a function of the spatial frequency in deg^−1^ and mm^−1^ chosen by the seven laboratories for the NPWE model.

## 4 DISCUSSION

This ILC demonstrated that Fourier‐based CT image quality metrics — TTF, NPS, and *d'* — are subject to substantial variability when computational methods are not standardized. For the NPW model, variability in *d′* resulted from the combined effects of differences in TTF and NPS measurements, mainly related to the ESF/LSF conditioning methods used for TTF computation and to ROI positioning for NPS measurement. The study could not separate their individual impacts. For the NPWE model, VTF and visualization parameters increased *d′* variability far beyond the effects seen from TTF and NPS differences alone. These findings are discussed in turn below.

It is advised that the CNR_T_, measured on the sum of all available images used for TTF calculation, should be at least 15 to achieve a reliable estimate of the TTF.^21^, ^30^ The CNR_T_ for the six series of images were well above this recommended value (Table 1), meaning that the differences between the TTFs of the seven laboratories did not stem from a lack of precision in the calculation. Different choices in the calculation methods highlighted in Table 2—ESF / LSF regression and/or windowing, ESF symmetrization and ESF fitting—lead to significant difference in TTFs, especially for the HR kernel. By minimizing manual user intervention, automated analysis approaches (iQmetrix‐CT, ImQuest) promised greater measurement accuracy and consistency, enhancing the efficiency of QC procedures.^13^, ^22^, ^42^ Nevertheless, in our study, L3 and L4 that used the same semi‐automated calculation software but with different options (Table 2) provided significantly different TTFs. Key techniques that include forcing the ESF to be strictly monotonic, mathematical ESF modeling using least squares fitting, and averaging multiple slices can be used to reduce the noise and improve the SNR in the ESF. In our study, analytic functions and interpolations lead to differences in TTFs, in particular certain information such as the overshoot related to edge enhancements (HR kernel) can be smoothed or poorly adjusted by parametric fits or a poor interpolation. Different pitches used for ESF/LSF interpolations also lead to differences in resulting TTFs between the laboratories. TTF from L2 was computed from an ESF that was interpolated with a sampling spacing of 100% the pixel size. This resulted in the TTF tending to zero as it approached the Nyquist frequency, a phenomenon not observed for HR kernel TTFs measured from oversampled interpolations of the ESF. Interpolation and fitting processes applied to reduce noise on the ESF must therefore be used carefully to keep an accurate representation, which is essential for subsequent TTF calculations. This is particularly important as the TTF of CT images resulting from non‐linear properties of IR or DLIR algorithms can depend on object contrast and noise level, so TTFs measurements need to be performed on images representing the imaging task under study, sometimes with a low CNR.^30^, ^43^, ^44^, ^45^ While techniques like logistical curve fitting are very effective at mitigating noise, averaging multiple images remains a robust, fundamental approach to obtain accurate, low‐noise data for TTF, especially in low‐contrast, high‐noise scenarios, and should be preferred for non‐linear IR and/or DLIR algorithms.

The laboratories measured similar NPS shapes (noise texture) except for L2 which measured a lower average NPS frequency *f_NPS_* (Table 4) for the HR series. Noise depends on the number of detected photons; fewer photons at the center of a homogeneous phantom mean higher noise. Bowtie filters balance photon flux, but strong central attenuation still leaves the center with fewer total counts relative to the periphery. In uniform CT images, this can lead to non‐uniform spatial distribution of noise, which therefore tends to increase toward the center and be minimal at the periphery.^24^ Differences in NPS measurement therefore primarily stemmed from the selection of the ROIs position and size in the images. Laboratory L1, which used a single small ROI at the center of the images (Figure 2), therefore provided the highest peak on the NPS curve. L2 and L7 positioned on the contrary their ROIs in the periphery of the images, resulting in lower NPS. L2 used an automated method developed for calculating NPS in patient images and that focuses on homogeneous regions in the patient body.^46^ The ROIs used were thus smaller (39 × 39 pixels) and focused on regions where the local noise was small, towards the border of the phantom. The other laboratories used single or multiple ROIs that covered different radial and angular positions to average the NPS over the images surface and produced similar results no matter which ROI size was used. L5 showed NPS curves with a low‐frequency increase that resulted from the low‐frequency trend of the HU values between the center and the periphery in the images. The other laboratories all removed this noise component by using different background subtraction techniques (2D polynomial or mean image subtraction), which gave similar results. Since the noise in CT images is not spatially stationary, a strict interpretation of the NPS as a full description of image noise is not possible, and the ROIs positions and size used for NPS calculation should be guided by a given task location, not specified to the laboratories in the present study. While smaller ROIs allow for local noise analysis, they require larger datasets to avoid high statistical uncertainty. These conclusions are consistent with those driven by previous studies,^31^, ^47^, ^48^ and highlight that NPS dependency to all these parameters should be considered for the interpretation of the NPS for task‐based image quality assessment.

Detectability was assessed with both NPW and NPWE observer models. The differences in VTF and visualization parameters dramatically increased the variability in the NPWE model. All the seven laboratories chose VTFs which had previously shown good agreement with human observer performance in previous CT studies.^14^, ^20^, ^40^, ^49^, ^50^ The sole consistent feature across these different VTFs is the peak near 4.0 deg^−^^1^, associated with human visual performance; however, this pattern was not observed for L1. Apart from this peak, there is no VTF standardization, and the visualization parameters (viewing distance and display magnification) deeply modified the frequency bandwidth of the VTF expressed in mm^−1^, and the resulting values for the NPWE model.^51^, ^52^, ^53^ In detectability studies using the human eye response, the absence of a standardized VTF necessitates the precise specification of all parameters to ensure valid comparisons and reduce variability in threshold detection. In the absence of a universal standard, inconsistent parameters can skew results. Regarding the decrease in detectability indices at 1.5 and 5.0 mGy compared to the values ​obtained at 15.0 mGy, the relative differences between laboratories remained below 10%. This allows for the consideration of using the NPWE model for a consistent calculation of relative detectability differences due to dose variations, beyond differences due to VTFs, which is essential for optimizing imaging protocols.

This study was limited to seven laboratories and a single CT system and was not intended to be exhaustive. Testing the same assessment tools on images acquired on different scanners would have required to repeat the study using a multi‐centric and multi‐scanner dataset to look for differences across all systems. This was beyond the scope of our study. Its objective was to show a quantitative comparison between a panel of laboratories that are used to assess image quality in computed tomography. An extension of the study to more or other laboratories, as well as to a larger number of CT scanners, could have yielded different quantitative results. The choice of another phantom and CT images with different resolution‐noise tradeoffs would have also exhibited different characteristics and quantitative results. Recommendations for image quality assessment in CT, focus on using standardized, phantom‐based measurements of TTF and NPS can nevertheless be made.

It is at first important to be aware that noticeable differences in TTF can be produced by ESF/LSF processing used to decrease the amount of noise in the data. In particular, fitting an analytical function to the ESF is very efficient but constrains the TTF to a shape that can be difficult to match the accurate form. Measuring the NPS in non‐homogeneous CT images is difficult because the standard assumption of a stationary (uniform) noise process is violated, making traditional Fourier‐based methods tricky. In heterogeneous patient anatomy, local noise characteristics change continuously, and anatomical structures interfere with the noise signature. Trying to compensate requires a complex and localized ROI analysis rather than simple global noise measurement. Different choices of ROIs guided by a given task location can therefore yield different NPS results. While there is no single universal standard for VTF parameters in the NPWE model for CT, studies generally use established models, such as the Burgess eye filter,^54^ or adapt them based on specific image display, viewer distance, and task conditions. Standard practice often involves customizing the NPWE model to match human observer responses for specific scanning conditions (kVp, pitch, slice thickness) rather than using a one‐size‐fits‐all parameter set. NPWE models are frequently “rescaled” or validated against human observer data using techniques like the Bland‐Altman method to ensure agreement, especially for varied scan protocols (e.g. different slice thicknesses, kernels).^55^, ^56^, ^57^ The trend is toward validation‐based optimization rather than fixed, rigid parameterization of the eye filter itself, ensuring the model's output aligns with human detection tasks in various clinical scenarios. For studies based on phantom images without direct human validation, it is therefore important to document the choice of all the parameters used to calculate *d′* values, which can be compared only if all the calculation parameters are identical.

It is worth noting that this study accounted for the variability driven by computational techniques acting upon inherently non‐stationary image features. A critical next step to effectively address variability in Fourier‐based evaluations of modern CT systems could involve modulating iterative reconstruction strength to quantify its direct influence on the variability of key performance metrics (TTF, NPS, and *d′*). This ILC shows nevertheless that it is necessary to standardize computational chains according to methodological recommendations. All dependencies should be analyzed and considered when interpreting image quality evaluation for a specific task. Standardization strategies could involve fixing standardized spatial locations and size of ROIs on uniform phantom modules to stabilize NPS estimation, adopting circular edge techniques without analytical fitting and excessive smoothing for TTF measurement, and enforcing a standardized mathematical description of the VTF across comparative evaluations to neutralize model‐inherent *d′* fluctuations in the NPWE model.

## 5 CONCLUSIONS

The interlaboratory comparative study demonstrated that evaluating CT image quality using TTF and NPS measurements on phantoms provided a comprehensive measure of spatial resolution and noise, allowing the derivation of simple detectability indices to classify image quality or scanner performance. The results also revealed important differences in TTF and NPS, with potential implications for measuring quantitative indices such as detectability. The study provided valuable insights into the sources of these quantitative differences between measurement methods. Software for the automated measurement of TTF, NPS and *d′* values on CT images has been developed with a view to standardization. The results highlighted incomplete agreement between laboratories, regardless of whether they used the same or different software. In the absence of complete standardization of image quality measurement, documentation of methods and all calculation parameters is essential. Concordance of detectability indices with human visual assessment is necessary to guarantee the reliability of measurements, particularly for variable acquisition protocols and viewing conditions.

## AUTHOR CONTRIBUTIONS

**Pascal Monnin**: Conceptualization; data curation; formal analysis; investigation; methodology; project administration; software; validation; visualization; writing—original draft. **Damien Racine**: Conceptualization; data curation; investigation; methodology; resources; validation; writing—review and editing. **Luis Ammour**: Data curation; formal analysis; resources; software; validation; writing—review and editing. **Yves Barbotteau**: Data curation; formal analysis; resources; software; validation; writing—review and editing. **Davide Cester**: Data curation; formal analysis; resources; software; validation; writing—review and editing. **Dimitar Petrov**: Data curation; formal analysis; resources; software; validation; writing—review and editing. **Kwinten Torfs**: Data curation; formal analysis; resources; software; validation; writing—review and editing. **Raffaelle Villa**: Data curation; formal analysis; resources; software; validation; writing—review and editing. **Nicoletta Paruccini**: Data curation; formal analysis; resources; software; validation; writing—review and editing. **Joël Greffier**: Data curation; formal analysis; resources; software; validation; writing—review and editing. **Anaïs Viry**: Conceptualization; data curation; methodology; project administration; resources; supervision; validation; visualization; writing—review and editing.

## CONFLICT OF INTEREST STATEMENT

The authors declare no conflicts of interest.

## Data Availability

The data that support the findings of this study are available from the corresponding author upon reasonable request.
